# Bisphenols alter thermal responses and performance in zebrafish (*Danio rerio*)

**DOI:** 10.1093/conphys/coaa138

**Published:** 2021-01-16

**Authors:** Nicholas C Wu, Frank Seebacher

**Affiliations:** School of Life and Environmental Sciences A08, The University of Sydney, New South Wales 2006, Australia

**Keywords:** Locomotion, metabolism, plastic pollution, temperature, thermal acclimation

## Abstract

Plastic pollutants are novel environmental stressors that are now persistent components of natural ecosystems. Endocrine disrupting chemicals such as bisphenols that leach out of plastics can modify physiological responses of animals by interfering with hormone signalling. Here, we tested whether three commonly produced bisphenols, bisphenol A (BPA), bisphenol F (BPF) and bisphenol S (BPS), impair thermal acclimation of swimming performance and metabolic enzyme [citrate synthase (CS) and lactate dehydrogenase (LDH)] activities in adult zebrafish (*Danio rerio*). We found that exposure to 30-μg l^−1^ BPF and BPS, but not BPA, reduced swimming performance, and no interactions between bisphenol exposure and acclimation (3 weeks to 18°C and 28°C) or acute test (18°C and 28°C) temperatures were found. BPA interacted with acclimation and acute test temperatures to determine CS activity, an indicator of mitochondrial density and aerobic metabolic capacity. BPS reduced CS activity and an interaction (at a one-tailed significance) between acclimation temperature and BPF exposure determined CS activity. LDH activity reflects anaerobic ATP production capacity, and BPA and BPF altered the effects of thermal acclimation and acute test temperatures on LDH activity. Our data show that all bisphenols we tested at ecologically relevant concentrations can disrupt the thermal responses of fish. BPS and BPF are used as environmentally safer alternatives to BPA, but our data show that these bisphenols are also of concern, particularly in thermally variable environments.

## Teaser

We show that bisphenols affect locomotion and metabolism and alter thermal acclimation of adult fish. Bisphenol pollution, therefore, can modify responses to thermal variation and climate change.

## Introduction

Endocrine disrupting chemicals (EDCs) that leach out of plastics or are used during plastic manufacture are of growing concern due to their global spread and environmental impact (Windsor *et al.*, 2018; Wu and Seebacher, 2020). Bisphenol A (BPA) is a common chemical plasticizer that can disrupt hormone signalling pathways in vertebrates, including glucocorticoids, oestrogen and thyroid hormone (Rubin, 2011; Rogers *et al.*, 2013). Consequently, BPA can affect animal growth, movement and behaviour (Yokota *et al.*, 2000; Aluru *et al.*, 2010; Little and Seebacher, 2015; Goundadkar and Katti, 2017). Because of its negative effects on wildlife and human health, BPA is increasingly replaced by alternatives such as bisphenol F (BPF) and bisphenol S (BPS) (Chen *et al.*, 2016). However, BPA replacements may have similar or more potent effects on animals (Tišler *et al.*, 2016; Moreman *et al.*, 2017; Park *et al.*, 2018; Zhang *et al.*, 2018).

In addition to their direct effects, EDCs can interfere with responses of animals to changing environments (DeCourten*et al.*, 2019). For example, thyroid hormone regulates thermal acclimation in zebrafish, particularly by increasing performance at low temperatures (Little *et al.*, 2013; Little and Seebacher, 2014). Exposure to BPA may inhibit the capacity to maintain physiological performance across temperature gradients (Little and Seebacher, 2015), since BPA and other bisphenols interfere with thyroid signalling (Moriyama*et al.*, 2002; Zhang *et al.*, 2018). Activities of enzymes, such as mitochondrial citrate synthase (CS) and glycolytic lactate dehydrogenase (LDH), reflect metabolic capacities and often acclimate to compensate for thermal variation (Guderley *et al.*, 2001; Seebacher *et al.*, 2003). Bisphenols can alter metabolic pathways in developing fish (Ortiz-Villanueva*et al.*, 2017), which is likely to be reflected in activities of CS and LDH. Similarly, locomotor performance is closely related to fitness (Le Galliard *et al.*, 2004) and, in many species, acclimates to different temperature regimes resulting in relatively stable performance despite temperature variation (Guderley, 2004). However, locomotor performance can be influenced by BPA in a temperature-dependent manner (Little and Seebacher, 2015). Understanding the potential disruption of thermal responses is especially important in the face of climate change because plastic pollution may interact with changing temperatures to modify animal physiology in hitherto unforeseen ways.

Our aim was to test whether different bisphenols alter thermal acclimation and acute thermal responses of locomotor performance and metabolic enzyme activities in zebrafish (*Danio rerio*). For each bisphenol (BPA, BPS and BPF), we conducted a fully factorial design testing the interactive effects of acclimation and acute test temperatures on locomotor performance, CS and LDH activities. We chose BPA, BPS and BPF because they are the three most common bisphenols found in aquatic environments (Wang *et al.*, 2017; Huang*et al.*, 2020; Wu and Seebacher, 2020). We hypothesized that (i) in the absence of bisphenol exposure, fish will show improved swimming performance and greater enzyme activities when acclimation and test temperatures match, compared with individuals not acclimated to that temperature; (ii) the exposure to bisphenols will diminish acclimation responses, so that compensation of performance for temperature variation is reduced; and (iii) the effects of bisphenol exposure will be greater at cold temperatures because it will attenuate the positive effect of thyroid hormone for cold acclimation. Zebrafish were used as a model system to understand the interactive effects of temperature and bisphenol, thus providing an important foothold to priorities research on organisms living in areas of high pollution and thermal fluctuation.

## Materials and methods

### Animal husbandry and experimental design

All experiments were approved by the University of Sydney Animal Ethics Committee (approval #2018/1139). Adult zebrafish were obtained from a commercial supplier (LiveFish, Bundaberg, Queensland, Australia) and maintained at 23.5 ± 0.3°C (thermal condition of hatchery) in aerated, aged water treated with stress coat (API Fishcare Inc., PA, USA) for a minimum of 1 week before experimentation. Before acclimation and bisphenol exposure trials, we measured critical sustained swimming speed (*U*_crit_; see below) of each experimental fish at two acute test temperatures (18°C and 28°C) with at least 24 h between trials (randomized between individuals). We took these preliminary measures to normalize data for variation between individuals. Each fish was then randomly allocated to one experimental group. We conducted a fully factorial experiment with bisphenol exposure [control (+vehicle) or exposed to either BPA, BPS or BPF], acclimation temperature (18°C and 28°C) and acute test temperature (18°C and 28°C) as factors. Fish were acclimated to their respective temperatures and exposed to bisphenols for 21 days before *U*_crit_ was measured again in the same individuals; as above, *U*_crit_ was measured in each fish at 18°C and 28°C (randomized between individuals) with a minimum of 24 h between measurements. We exposed fish to 30 μg l^−1^ of each bisphenol [BPA (≥99%), Sigma Aldrich, Castle Hill, Australia; BPS (≥98%), Sigma Aldrich; BPF (>99), Tokyo Chemical Industry, Tokyo, Japan]. BPA concentrations of 30 μg l^−1^ are present in natural aquatic systems (Wu and Seebacher, 2020), while the levels of BPS and BPF are expected to rise in natural water bodies as BPA is phased out of plastic production. The control treatments were exposed to 0.005% v/v of ethanol, which was used as the vehicle control. Water temperatures in the study represent the natural thermal ranges zebrafish experience in the wild (Engeszer *et al.*, 2007). Water temperature was monitored (every 30 min) with water temperature loggers (HOBO MX2201, OneTemp Pty Ltd, Adelaide, Australia) throughout the acclimation period and was maintained within 0.5°C of the desired acclimation temperatures. Within each treatment, there were three replicate tanks (8 l) containing five zebrafish. Within the tanks, each fish was housed individually in a 1-l cylindrical basket that permitted water flow, and hence visual and chemical contact between fish, but prevented fish from leaving so that individuals could be tracked for *U*_crit_ measurements before and after experimental treatments. The light cycle was 14-h light:10-h dark. Fish were fed with fish flakes (Tetramin, VA, USA) until satiated, six times per week, and a 50% water change was performed three times a week. We replenished bisphenol and vehicle controls after cleaning tanks to maintain the desired nominal concentrations.

### Critical sustained swimming speed

Fish critical sustained swimming speed (*U*_crit_) was determined by an incremental exercise trial (in *n* = 9–15 fish per treatment) according to published protocols (Little and Seebacher, 2015). Briefly, *U*_crit_ was measured in a clear plastic (Perspex) flume (210-mm length and 26-mm internal diameter) fitted over the intake end of an inline submersible pump (iL500P 12 V, Rule, Miami, FL, USA), which drew water through the flume. Hollow straws at the inlet end of the flume helped maintain laminar flow. The flume and pump were submerged in a tank (59 × 37 × 26 cm). A DC power supply (NP-9615, Manson Engineering Industrial, Hong Kong) was used to adjust the flow rate, which was monitored in real-time with a flowmeter (DigiFlow 6710M-66, Savant Electronics Inc., Taichung, Taiwan) connected to the outlet of each pump. After introduction into the flume, fish swam at 0.1 m s^−1^ for 10 min after which flow speed was increased by 0.06 m s^−1^ (${U}_i$) every 10 min (${T}_i$) until fish could no longer hold their position in the water flow. The first time a fish stopped swimming and fell back onto the grid dividing the flume from the pump, the flow was stopped for 5–10 s, after which it was increased again to the previous setting. The second time a fish stopped swimming, the trial was terminated and the time until exhaustion was recorded. *U*_crit_ (m s^−1^) was calculated as

\begin{equation*} U_{crit} ={U}_f+\left(\frac{T_f}{T_i}\right)\times{U}_i, \end{equation*}

where ${U}_f$ is the highest velocity maintained for the entire swim interval and ${T}_f$ is the time to exhaustion in the final speed interval. Fish were fasted for 24 h before the experiment. The body mass (g) and the standard length (cm) were determined after the completion of the *U*_crit_ trial. Standard length (tip of the snout to the posterior end of the last vertebrae, excluding tail fin) was measured by photographing the fish in a transparent container (64 × 40 × 16 mm) with a white background on one side and markers on the bottom spaced out at 1 cm each. Photos were analysed with the ImageJ (Schneider *et al.*, 2012) where the image was calibrated with the markers. Body mass was measured by gentle patting down the fish (while in the soft mesh net) with paper towels before placing on a tared scale with a container to hold the fish (mean ± SD mass = 0.46 ± 0.09 g and length = 2.98 ± 0.18 cm).

**Figure 1:**
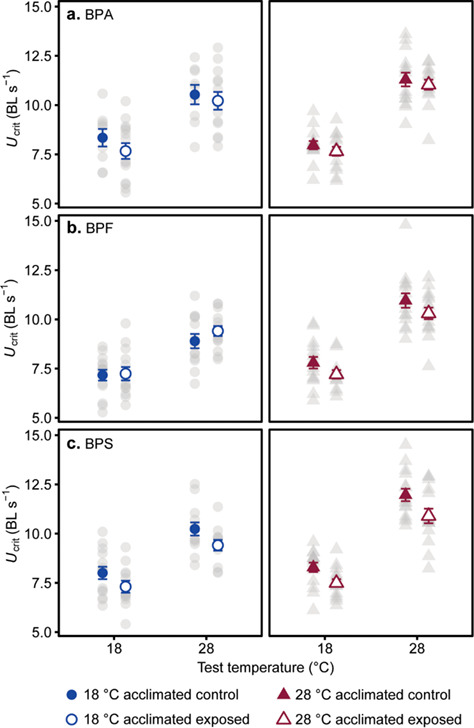
Effect of acclimation temperature and bisphenol exposure on swimming speed (*U*_crit_). Bisphenol A (a) exposure did not have a significant effect on *U*_crit_ measured at 18°C and 28°C acute test temperatures in fish acclimated to 18°C and 28°C, but bisphenol F (b) and bisphenol S (c) exposure significantly reduced *U*_crit_. Individual data points (grey circles = 18°C acclimated and grey triangles = 28°C acclimated) as well as means ± s.e. (blue circles = 18°C acclimated and red triangles = 28°C acclimated) are shown. Samples sizes were 9–15 fish per treatment group. A full statistical summary is provided in Table S1 and relative change in *U*_crit_ from pre-exposure presented in Fig. S1.

**Figure 2:**
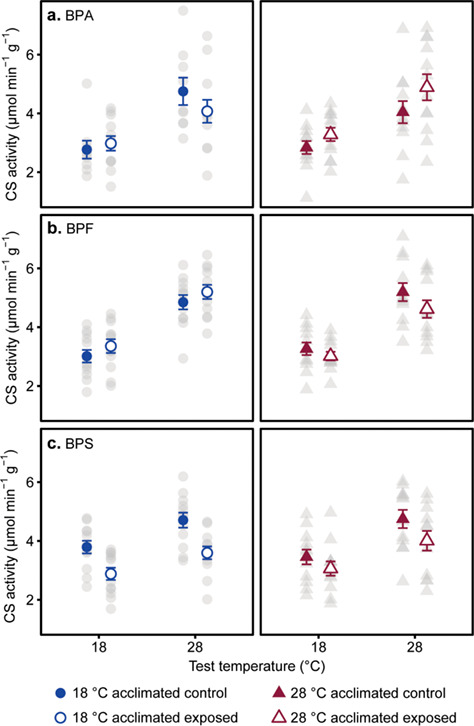
Effect of acclimation temperature and bisphenol exposure on CS activity (μmol min^−1^ g^−1^). Bisphenol A (a) exposure showed a three-way interaction with test temperature and acclimated temperature. Bisphenol F (b) exposure showed a two-way interaction with acclimated temperature. Bisphenol S (c) exposure reduced CS activity across all test and acclimated temperature. Individual data points (grey circles = 18°C acclimated and grey triangles = 28°C acclimated) as well as means ± s.e. (blue circles = 18°C acclimated and red triangles = 28°C acclimated) are shown. Samples sizes were 9–12 fish per treatment group. A full statistical summary is provided in Table S2.

### Metabolic enzyme assays

After measuring *U*_crit_, fish were euthanized by anaesthesia in 250 μL l^−1^ of AQUI-S (Lower Hutt, New Zealand) followed by cervical dislocation. Tail and rostral muscle were dissected and immediately frozen in liquid nitrogen and stored at −80°C. Enzyme assays were conducted according to published protocols (Thibault *et al.*, 1997). Muscle samples were homogenized (in a TissueLyser LT, Qiagen, Melbourne, Australia) in nine volumes of extraction buffer (50-mM imidazole, 2-mM MgCl₂, 5-mM EDTA, 0.1% Triton, 1-mM glutathione at pH 7.5; all chemicals from Sigma Aldrich, Castle Hill, Australia). For LDH assays, the homogenate was further diluted to 1:100 (for 18°C test temperature) and 1:200 (for 28°C test temperature) in the extraction buffer. CS activity (*n* = 12 fish per treatment) was measured as the reduction of DTNB [5,5′ dithiobis-(2-nitrobenzoic) acid]. The assay was conducted in a 100-mmol Tris buffer (pH = 8) containing 0.1-mM DTNB, 0.1-mM acetyl CoA and 15-mM oxaloacetate. We conducted control assays without addition of substrate (oxaloacetate) to check for reduction of DTNB other than by CS. LDH activity (*n* = 12 fish per treatment) was measured as the reduction of NADH in a potassium phosphate (KH_2_PO_4_/K_2_PO_4_; 100 mM) buffer at pH 7, with 0.16-mM NADH and 0.4-mM sodium pyruvate. Enzyme activities were measured as a change in absorbance (412 or 340 nm for CS and LDH, respectively) in a UV/visible spectrophotometer (Ultrospec 2100 Pro; GE Healthcare, Sydney, Australia) with a temperature-controlled cuvette holder. Assays were carried out in duplicate at the acute test temperatures of 18°C and 28°C. Absorbance was measured for 3 min and activity was expressed as μmol of substrate converted min^−1^ g^−1^ wet tissue.

### Statistical analysis

We analysed data with linear mixed effects (lme) models, which were performed with the ‘lmer’ function in the *lmerTest* package (Kuznetsova *et al.*, 2017) in *R* (R Core Team, 2018), and the estimated marginal means were computed with the ‘emmeans’ function from the *emmeans* package (Lenth *et al.*, 2020). To examine the effect of BPA, BPF and BPS on the thermal acclimation response of *U*_crit_ (m s^−1^) and CS and LDH activities, lme models were analysed with a three-way interaction of acclimation temperature, bisphenol exposure and acute test temperature as predictor variables and using body length and pre-treatment *U*_crit_ as covariates (for *U*_crit_ only). Fish identity was included as a random effect to account for repeated measurements at different acute test temperature. Data were visually presented as mean ± standard error (s.e.).

## Results

### Swimming performance

In all bisphenol treatments, *U*_crit_ increased with increasing test temperature (main effects of acute temperature; Table 1) and there was an interaction between acclimation temperature and acute temperature; fish acclimated at 28°C had a higher *U*_crit_ when measured at 28°C acute temperature than fish acclimated to 18°C and measured at 28°C acute temperature. There was no significant effect of BPA exposure on *U*_crit_ (Fig. 1a), but exposures to BPF and BPS caused significant reductions in *U*_crit_ (main effects; Fig. 1b and c; Table 1). There were no significant interactions between bisphenol exposure and acclimation or acute test temperatures (two- and three-way interactions; Table 1).

**Table 1:** Statistical results for analysis of swimming performance (*U*_crit_)

	BPA		BPF		BPS	
	F_df_	*P*	F_df_	*P*	F_df_	*P*
U_crit_ (pre)	0.02_1,78_	0.9	45.3_1,102_	<0.0001	50.0_1,97_	<0.0001
Body length	4_1,49_	<0.05	2.3_1,102_	0.1	5.3_1,50_	0.02
AccT	1.6_1,50_	0.2	24.9_1,102_	<0.0001	19.5_1,50_	<0.0001
Exp	0.9_1,46_	0.4	8.7_1,102_	0.004	13.3_1,50_	<0.0001
TestT	67.2_1,72_	<0.0001	12.7_1,102_	0.0005	26.7_1,86_	<0.0001
AccT x Exp	0.2_1,48_	0.7	1.6_1,102_	0.2	0.4_1,50_	0.5
AccT x TestT	15.2_1,46_	0.0003	5.8_1,102_	0.01	21.7_1,50_	< 0.0001
Exp x TestT	0.8_1,46_	0.4	0.3_1,102_	0.6	0.8_1,50_	0.4
AccT x Exp x TestT	0.008_1,46_	0.9	0.3_1,102_	0.56	0.03_1,50_	0.6

F-values with degrees of freedom (as subscripts) and P-values are shown for main effects and interactions between acclimation temperature (AccT), bisphenol exposure (Exp) and acute test temperature (TestT).

Pre-exposure *U*_crit_ (pre) and body length were covariates.

### CS activity

In all bisphenol treatments, CS activity increased with increasing test temperature (main effects; Table 2). There was a three-way interaction between BPA exposure, acclimation temperature and acute temperature. At 28°C test temperature, BPA decreased CS activity in cold acclimated fish but increased it in warm acclimated fish, while at 18°C test temperature, there was an increase in CS activity regardless of acclimation temperature (Fig. 2a). Exposure to BPF resulted in an interaction between acclimation temperature and exposure at *P* = 0.08, where BPF increased CS activity in cold acclimated animals but decreased it in warm acclimated fish (Fig. 2b). BPS caused a reduction in CS activity regardless of temperature (main effect; Table 2; Fig. 2c). There were no significant interactions between BPS exposure, acclimation temperature and acute test temperature influencing CS activity (two- and three-way interactions; Table 2).

**Table 2:** Statistical results for analysis of CS activity

	BPA		BPF		BPS	
	F_df_	*P*	F_df_	*P*	F_df_	*P*
AccT	0.14_1,41_	0.7	0.15_1,44_	0.7	0.11_1,44_	0.74
Exp	1.42_1,41_	0.52	0.02_1,44_	0.87	12.47_1,44_	0.0009
TestT	199_1,41_	<0.0001	239_1,44_	<0.0001	56.4_1,44_	<0.0001
AccT x Exp	1.9_1,41_	0.17	3.19_1,44_	0.08	0.97_1,44_	0.32
AccT x TestT	0.24_1,41_	0.62	0.1_1,44_	0.75	1.39_1,44_	0.24
Exp x TestT	0.81_1,41_	0.37	0.49_1,44_	0.49	1.14_1,44_	0.29
AccT x Exp x TestT	5.7_1,41_	0.02	0.52_1,44_	0.47	0.07_1,44_	0.78

F-values with degrees of freedom (as subscripts) and P-values are shown for main effects and interactions between AccT, Exp and TestT.

### LDH activity

**Figure 3:**
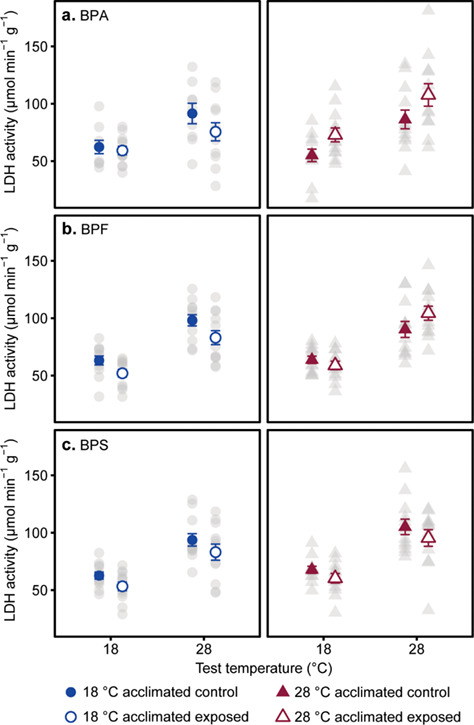
Effect of acclimation temperature and bisphenol exposure on the LDH activity (μmol min^−1^ g^−1^). Bisphenol A (a) exposure showed a two-way interaction with acclimated temperature. Bisphenol F (b) exposure showed a three-way interaction with test temperature and acclimated temperature. Bisphenol S (c) exposure reduced LDH activity across all test and acclimated temperature. Individual data points (grey circles = 18°C acclimated and grey triangles = 28°C acclimated) as well as means ± s.e. (blue circles = 18°C acclimated and red triangles = 28°C acclimated) are shown. Samples sizes were 9–12 fish per treatment group. A full statistical summary is provided in Table S3.

As for *U*_crit_ and CS activity, LDH activity increased with increasing test temperature in all bisphenol treatments (main effects; Table 3). There was a significant interaction between acclimation temperature and BPA exposure, and BPA decreased LDH activity in cold-acclimated fish but increased it in warm-acclimated fish (Fig. 3a). A three-way interaction indicates that BPF exposure decreased LDH activity in all treatments, except in warm acclimated fish at 28°C acute test temperature where BPF increased LDH activity (Fig. 3b). BPS decreased LDH activity across all temperature treatments at *P* = 0.05 (main effect; Table 3; Fig. 3c). There were no significant interactions between BPS exposure and acclimation or test temperature that influences LDH activity (two- and three-way interactions; Table 3).

**Table 3:** Statistical results for analysis of LDH activity

	BPA		BPF		BPS	
	F_df_	*P*	F_df_	*P*	F_df_	*P*
AccT	1.69_1,41_	0.19	1.6_1,44_	0.21	3.55_1,44_	0.06
Exp	0.62_1,41_	0.43	1.08_1,44_	0.3	3.8_1,44_	0.05
TestT	61.9_1,41_	<0.0001	153_1,44_	<0.0001	195_1,44_	<0.0001
AccT x Exp	5.19_1,41_	0.02	4.7_1,44_	0.03	0.02_1,44_	0.87
AccT x TestT	2.12_1,41_	0.15	0.29_1,44_	0.59	1.49_1,44_	0.22
Exp x TestT	0.44_1,41_	0.5	1.18_1,44_	0.18	0.14_1,44_	0.07
AccT x Exp x TestT	1.34_1,41_	0.25	4.26_1,44_	0.04	0.01_1,44_	0.91

F-values with degrees of freedom (as subscripts) and P-values are shown for main effects and interactions between AccT, Exp and TestT.

## Discussion

The ubiquitous presence of bisphenols in the environment provides a novel challenge for organisms. Our results show that at ecologically relevant concentrations, bisphenols disrupted swimming performance and altered metabolic enzyme activities. The effects of BPA and BPF on CS and LDH activities changed in magnitude and direction depending on the combination of chronic acclimation and acute temperatures. This temperature-specificity is important because it shows that bisphenols alter the way in which animals respond to other environmental drivers. In contrast, BPS had a negative effect on all response variables regardless of temperature.

We hypothesized that bisphenol exposure would diminish acclimation capacity. Fish exposed to bisphenols at low test temperatures would show a decrease in *U*_crit_, and the effects would be greater in cold-acclimated animals. We did not observe an interactive effect of bisphenol exposure with either acclimation or acute test temperature on swimming performance. However, BPS and BPF disrupted *U*_crit_ to a greater extent than BPA. The bisphenol-specific effects may relate to the differences in binding affinity to nuclear receptors (Usman and Ahmad, 2019), which can alter receptor sensitivity and compatibility, and the differences in metabolic pathway disruption (Zhang *et al.*, 2018; Qiu *et al.*, 2019). BPF and BPS are less toxic than BPA and cause lower rates of mortality and deformity in zebrafish embryos and larvae than BPA (Moreman *et al.*, 2017), which prompted the use of BPF and BPS as safer alternatives for plastic manufacturing. However, our data show that BPF and BPS had significantly greater effects on adult swimming performance, which can have lasting ecological consequences and reduce fitness (Husak, 2016). Future studies should consider how bisphenol concentrations change with temperature over time.

Muscle enzyme activity can be an important indicator of fitness, and glycolytic and mitochondrial enzyme activities are linked positively to swimming performance and the energetic status of animals (Guderley *et al.*, 2001; Martinez *et al.*, 2003; Guderley, 2004). Reduced enzyme activities and muscle function can increase susceptibly to predation and decrease reproductive success (Le Galliard *et al.*, 2004; Husak *et al.*, 2006). Mechanistically, our results may be explained by disrupted thyroid signalling. Bisphenols bind to thyroid hormone receptors, inhibiting 3,3′,5-triiodothyronine (T3) and 3,5-diiodothyronine (T2) actions (Moriyama *et al.*, 2002; Gentilcore *et al.*, 2013). Thyroid hormones are important for regulating metabolism, muscle function, growth and development (Mullur *et al.*, 2014). Disruption of thyroid function from bisphenol exposure can therefore interfere with locomotor performance and energy metabolism (Casals-Casas and Desvergne, 2011; Little *et al.*, 2013). However, determining if changes in swimming performance and activities of metabolic enzymes are directly due to thyroid disruption requires thyroid manipulation or quantifying activity of thyroid receptors in response to bisphenol exposure for future studies. In addition to thyroid disruption, bisphenols could disrupt glucocorticoid receptors, which could interfere with HPA axis signalling and metabolism (MacKay and Abizaid, 2018; Guindre-Parker, 2020), and thereby affect growth and reproduction (Schreck, 2010; Jerez-Cepa *et al.*, 2019).

We found that BPA and BPF altered mitochondrial and glycolytic enzyme activities in a temperature-dependent manner. Bisphenol exposure can therefore alter the way in which animals respond to environmental temperature variation. While BPF and BPS have similar endocrine disrupting effects as BPA (Héliès-Toussaint *et al.*, 2014; Rochester and Bolden, 2015), our study emphasizes the complex nature of inferring the disruptive effects of endocrine disruptors because they can change depending on the environmental conditions. Chemical exposure and physiological acclimation can change the sensitivity of different receptors (Casals-Casas and Desvergne, 2011; Segner *et al.*, 2014), which means that the mechanisms underlying bisphenol toxicity at one temperature may not reflect those that mediate its toxicity at a different temperature. Temperature can also influence the rate of bisphenol uptake and excretion, resulting in differences in bioaccumulation (Heinonen *et al.*, 2002; Honkanen and Kukkonen, 2006). Together, thermally dependent bioaccumulation rates and temperature-sensitive effects emphasize that EDCs need to be studied under ecologically relevant conditions and projected climate change scenarios to provide an accurate representation of the effects of plastic pollution in a changing world.

Of particular concern are global ‘hotspots’ where high rates of predicted warming coincide with high concentrations of bisphenol pollution. For example, tropical regions in Africa, South America and South Asia are predicted to warm at a disproportionally high rate (Malcolm *et al.*, 2006; King and Harrington, 2018) and also experienced high levels of bisphenol pollution (e.g. BPA; Wu and Seebacher, 2020). Tropical regions also have high biodiversity, so that improving management of plastic pollution and production is particularly urgent in these regions. Our study also indicated that bisphenols alter the manifestation of thermal plasticity (acclimation), thereby altering the compensatory capacity of animals to respond to thermal change. With respect to climate warming in particular, BPF decreased CS activity at warm acclimation (at a one-tailed significance) and test temperatures, while it increased LDH activity under these conditions. These results indicate that BPF exposure may decrease oxidative metabolic capacity and increase reliance on anaerobic metabolism as climates warm, which would have substantial negative consequences for growth and activity.

Understanding how natural populations respond to climate change requires consideration of how pollutants alter thermal plasticity because thermal plasticity of physiological processes is likely to be a key factor in determining species resilience to climate change (Seebacher *et al.*, 2015). Additionally, early life stages of aquatic organisms are more sensitive to bisphenols than adults (Wu and Seebacher, 2020), and in future research, it is important to consider how early life exposure to EDCs could affect developmental acclimation to thermal stressors. Pollution is now a permanent component of the natural environment, and various sources of pollution such as agricultural and urban run-offs (Morgan *et al.*, 2001; Lannig *et al.*, 2006; Isaza *et al.*, 2020), dam-induced cold-water pollution (Parisi *et al.*, 2020) and light pollution (Noyes and Lema, 2015) can interact to affect how organism respond to temperature. We therefore highlight the need for experimental biologists, toxicologists and conservation management to incorporate pollution into climate studies to provide more realistic estimates of responses to warming in aquatic, and possibly terrestrial, organisms.

## Supplementary Material

CONPHYS-2020-161_-_SI_coaa138

## Data Availability

All datasets generated and analysed during the study is available on the GitHub repository: https://github.com/nicholaswunz/EDC-temp-performance.
