# Supplementary material for: Bisphenols alter thermal responses and performance in zebrafish (Danio rerio)
Source: Conserv Physiol. 2021 Jan 16;9(1):coaa138. doi: 10.1093/conphys/coaa138 (PMC7816798; doi:10.1093/conphys/coaa138)
Supplement: CONPHYS-2020-161_-_SI_coaa138 [file conphys-2020-161_-_si_coaa138.docx]

# Bisphenols alter thermal responses and performance in zebrafish (*Danio rerio*)

Nicholas C. Wu, and Frank Seebacher^*^

*School of Life and Environmental Sciences A08, The University of Sydney, NSW 2006, Australia*

# SUPPLEMENTARY INFORMATION

## Supplementary tables

Table S1. Summary statistics from the *U*_crit_ estimated marginal means pairwise comparison for BPA, BPF, BPS. Names were ordered as acclimation temperature (18/28 °C), exposure (control/exposed), and acute temperature (18/28 °C).

| **BPA** | | | | | |
| --- | --- | --- | --- | --- | --- |
| **accl temp, exposure, acute temp** | **estimate** | **SE** | **df** | **t.ratio** | **p.value** |
| 18,control,18 - 28,control,18 | 0.01 | 0.02 | 69.6 | 0.55 | 1.00 |
| 18,control,18 - 18,BPA,18 | 0.02 | 0.02 | 66.3 | 1.09 | 0.96 |
| 18,control,18 - 28,BPA,18 | 0.02 | 0.02 | 66.9 | 1.06 | 0.96 |
| 18,control,18 - 18,control,28 | -0.06 | 0.01 | 61 | -4.53 | 0.00 |
| 18,control,18 - 28,control,28 | -0.09 | 0.02 | 73.2 | -5.32 | <.0001 |
| 18,control,18 - 18,BPA,28 | -0.05 | 0.02 | 79.1 | -3.05 | 0.06 |
| 18,control,18 - 28,BPA,28 | -0.09 | 0.02 | 75 | -5.05 | 0.0001 |
| 28,control,18 - 18,BPA,18 | 0.01 | 0.01 | 67.8 | 0.61 | 1.00 |
| 28,control,18 - 28,BPA,18 | 0.01 | 0.01 | 66.3 | 0.61 | 1.00 |
| 28,control,18 - 18,control,28 | -0.07 | 0.02 | 91.5 | -3.48 | 0.02 |
| 28,control,18 - 28,control,28 | -0.10 | 0.01 | 65.1 | -7.88 | <.0001 |
| 28,control,18 - 18,BPA,28 | -0.06 | 0.02 | 91.4 | -3.49 | 0.02 |
| 28,control,18 - 28,BPA,28 | -0.10 | 0.02 | 89.4 | -5.59 | <.0001 |
| 18,BPA,18 - 28,BPA,18 | 0.00 | 0.01 | 66.1 | -0.02 | 1.00 |
| 18,BPA,18 - 18,control,28 | -0.08 | 0.02 | 87.1 | -4.14 | 0.002 |
| 18,BPA,18 - 28,control,28 | -0.11 | 0.02 | 80.4 | -6.84 | <.0001 |
| 18,BPA,18 - 18,BPA,28 | -0.07 | 0.01 | 64.4 | -5.70 | <.0001 |
| 18,BPA,18 - 28,BPA,28 | -0.11 | 0.02 | 83.3 | -6.50 | <.0001 |
| 28,BPA,18 - 18,control,28 | -0.08 | 0.02 | 88.9 | -4.00 | <.0001 |
| 28,BPA,18 - 28,control,28 | -0.11 | 0.02 | 83.6 | -6.65 | <.0001 |
| 28,BPA,18 - 18,BPA,28 | -0.07 | 0.02 | 88.9 | -4.11 | 0.002 |
| 28,BPA,18 - 28,BPA,28 | -0.10 | 0.01 | 64.7 | -8.53 | <.0001 |
| 18,control,28 - 28,control,28 | -0.03 | 0.02 | 69.8 | -1.57 | 0.77 |
| 18,control,28 - 18,BPA,28 | 0.01 | 0.02 | 68.2 | 0.57 | 1.00 |
| 18,control,28 - 28,BPA,28 | -0.02 | 0.02 | 67.4 | -1.48 | 0.82 |
| 28,control,28 - 18,BPA,28 | 0.03 | 0.01 | 69.1 | 2.46 | 0.23 |
| 28,control,28 - 28,BPA,28 | 0.00 | 0.01 | 66.1 | 0.11 | 1.00 |
| 18,BPA,28 - 28,BPA,28 | -0.03 | 0.01 | 67.7 | -2.36 | 0.28 |
| **BPF** | | | | | |
| **accl temp, exposure, acute temp** | **estimate** | **SE** | **df** | **t.ratio** | **p.value** |
| 18,control,18 - 28,control,18 | -0.02 | 0.01 | 102 | -1.75 | 0.66 |
| 18,control,18 - 18,BPS,18 | 0.01 | 0.01 | 101.4 | 0.86 | 0.99 |
| 18,control,18 - 28,BPS,18 | 0.00 | 0.01 | 102 | -0.06 | 1.00 |
| 18,control,18 - 18,control,28 | -0.02 | 0.01 | 68.9 | -1.49 | 0.81 |
| 18,control,18 - 28,control,28 | -0.07 | 0.01 | 101.4 | -5.39 | <.0001 |
| 18,control,18 - 18,BPS,28 | -0.01 | 0.01 | 100.7 | -0.68 | 1.00 |
| 18,control,18 - 28,BPS,28 | -0.04 | 0.01 | 101.2 | -2.87 | 0.09 |
| 28,control,18 - 18,BPS,18 | 0.03 | 0.01 | 101.7 | 2.57 | 0.18 |
| 28,control,18 - 28,BPS,18 | 0.02 | 0.01 | 102 | 1.66 | 0.71 |
| 28,control,18 - 18,control,28 | 0.00 | 0.01 | 101.9 | 0.02 | 1.00 |
| 28,control,18 - 28,control,28 | -0.05 | 0.01 | 73.8 | -3.96 | <.0001 |
| 28,control,18 - 18,BPS,28 | 0.01 | 0.01 | 101.1 | 0.73 | 1.00 |
| 28,control,18 - 28,BPS,28 | -0.02 | 0.01 | 101.3 | -1.53 | 0.79 |
| 18,BPS,18 - 28,BPS,18 | -0.01 | 0.01 | 101.3 | -0.91 | 0.99 |
| 18,BPS,18 - 18,control,28 | -0.03 | 0.01 | 101.2 | -2.20 | 0.36 |
| 18,BPS,18 - 28,control,28 | -0.08 | 0.01 | 100.7 | -6.01 | <.0001 |
| 18,BPS,18 - 18,BPS,28 | -0.02 | 0.01 | 71.2 | -1.46 | 0.83 |
| 18,BPS,18 - 28,BPS,28 | -0.05 | 0.01 | 100.5 | -3.51 | 0.01 |
| 28,BPS,18 - 18,control,28 | -0.02 | 0.01 | 101.7 | -1.42 | 0.85 |
| 28,BPS,18 - 28,control,28 | -0.07 | 0.01 | 101.2 | -5.28 | <.0001 |
| 28,BPS,18 - 18,BPS,28 | -0.01 | 0.01 | 100.2 | -0.63 | 1.00 |
| 28,BPS,18 - 28,BPS,28 | -0.04 | 0.01 | 83.2 | -2.80 | 0.11 |
| 18,control,28 - 28,control,28 | -0.05 | 0.01 | 102 | -4.66 | 0.0002 |
| 18,control,28 - 18,BPS,28 | 0.01 | 0.01 | 101.5 | 0.80 | 0.99 |
| 18,control,28 - 28,BPS,28 | -0.02 | 0.01 | 101.9 | -1.89 | 0.56 |
| 28,control,28 - 18,BPS,28 | 0.06 | 0.01 | 101.5 | 5.52 | <.0001 |
| 28,control,28 - 28,BPS,28 | 0.03 | 0.01 | 102 | 2.84 | 0.10 |
| 18,BPS,28 - 28,BPS,28 | -0.03 | 0.01 | 101.5 | -2.76 | 0.12 |
| **BPS** | | | | | |
| **accl temp, exposure, acute temp** | **estimate** | **SE** | **df** | **t.ratio** | **p.value** |
| 18,control,18 - 28,control,18 | -0.01 | 0.01 | 83 | -1.18 | 0.93 |
| 18,control,18 - 18,BPF,18 | 0.02 | 0.01 | 82.7 | 1.63 | 0.73 |
| 18,control,18 - 28,BPF,18 | 0.01 | 0.01 | 83.3 | 0.95 | 0.98 |
| 18,control,18 - 18,control,28 | -0.02 | 0.01 | 68.8 | -2.21 | 0.36 |
| 18,control,18 - 28,control,28 | -0.07 | 0.01 | 94 | -6.13 | <.0001 |
| 18,control,18 - 18,BPF,28 | 0.00 | 0.01 | 90.1 | -0.13 | 1.00 |
| 18,control,18 - 28,BPF,28 | -0.04 | 0.01 | 93.9 | -3.44 | 0.02 |
| 28,control,18 - 18,BPF,18 | 0.03 | 0.01 | 82.1 | 2.76 | 0.12 |
| 28,control,18 - 28,BPF,18 | 0.02 | 0.01 | 83.1 | 2.17 | 0.38 |
| 28,control,18 - 18,control,28 | -0.01 | 0.01 | 94.5 | -0.81 | 0.99 |
| 28,control,18 - 28,control,28 | -0.06 | 0.01 | 72.4 | -6.15 | <.0001 |
| 28,control,18 - 18,BPF,28 | 0.01 | 0.01 | 90.5 | 0.86 | 0.99 |
| 28,control,18 - 28,BPF,28 | -0.03 | 0.01 | 94.7 | -2.43 | 0.24 |
| 18,BPF,18 - 28,BPF,18 | -0.01 | 0.01 | 82.5 | -0.74 | 1.00 |
| 18,BPF,18 - 18,control,28 | -0.04 | 0.01 | 93.7 | -3.09 | 0.05 |
| 18,BPF,18 - 28,control,28 | -0.09 | 0.01 | 93.8 | -7.14 | <.0001 |
| 18,BPF,18 - 18,BPF,28 | -0.02 | 0.01 | 64.8 | -1.92 | 0.54 |
| 18,BPF,18 - 28,BPF,28 | -0.06 | 0.01 | 93.8 | -4.62 | 0.0003 |
| 28,BPF,18 - 18,control,28 | -0.03 | 0.01 | 95.3 | -2.55 | 0.19 |
| 28,BPF,18 - 28,control,28 | -0.08 | 0.01 | 95.7 | -6.77 | <.0001 |
| 28,BPF,18 - 18,BPF,28 | -0.01 | 0.01 | 91.7 | -0.92 | 0.98 |
| 28,BPF,18 - 28,BPF,28 | -0.05 | 0.01 | 73.5 | -4.95 | 0.0001 |
| 18,control,28 - 28,control,28 | -0.05 | 0.01 | 84.9 | -5.08 | 0.0001 |
| 18,control,28 - 18,BPF,28 | 0.02 | 0.01 | 84.4 | 1.85 | 0.59 |
| 18,control,28 - 28,BPF,28 | -0.02 | 0.01 | 84.9 | -1.89 | 0.56 |
| 28,control,28 - 18,BPF,28 | 0.07 | 0.01 | 82.8 | 6.73 | <.0001 |
| 28,control,28 - 28,BPF,28 | 0.03 | 0.01 | 83 | 3.31 | 0.03 |
| 18,BPF,28 - 28,BPF,28 | -0.04 | 0.01 | 83.1 | -3.71 | 0.01 |

Table S2. Summary statistics from the CS activity estimated marginal means pairwise comparison for BPA, BPF, BPS. Names were ordered as acclimation temperature (18/28 °C), exposure (control/exposed), and acute temperature (18/28 °C).

| **BPA** | | | | | |
| --- | --- | --- | --- | --- | --- |
| **contrast** | **estimate** | **SE** | **df** | **t.ratio** | **p.value** |
| 18,control,18 - 28,control,18 | -0.07 | 0.51 | 55.3 | -0.14 | 1.00 |
| 18,control,18 - 18,BPA,18 | -0.21 | 0.51 | 55.3 | -0.42 | 1.00 |
| 18,control,18 - 28,BPA,18 | -0.51 | 0.51 | 55.3 | -1.02 | 0.97 |
| 18,control,18 - 18,control,28 | -1.98 | 0.30 | 41 | -6.63 | <.0001 |
| 18,control,18 - 28,control,28 | -1.27 | 0.51 | 55.3 | -2.52 | 0.21 |
| 18,control,18 - 18,BPA,28 | -1.30 | 0.51 | 55.3 | -2.57 | 0.19 |
| 18,control,18 - 28,BPA,28 | -2.12 | 0.51 | 55.3 | -4.18 | 0.003 |
| 28,control,18 - 18,BPA,18 | -0.14 | 0.47 | 55.3 | -0.30 | 1.00 |
| 28,control,18 - 28,BPA,18 | -0.44 | 0.47 | 55.3 | -0.95 | 0.98 |
| 28,control,18 - 18,control,28 | -1.91 | 0.51 | 55.3 | -3.77 | 0.01 |
| 28,control,18 - 28,control,28 | -1.20 | 0.26 | 41 | -4.65 | 0.00 |
| 28,control,18 - 18,BPA,28 | -1.23 | 0.47 | 55.3 | -2.63 | 0.17 |
| 28,control,18 - 28,BPA,28 | -2.05 | 0.47 | 55.3 | -4.37 | 0.001 |
| 18,BPA,18 - 28,BPA,18 | -0.30 | 0.47 | 55.3 | -0.65 | 1.00 |
| 18,BPA,18 - 18,control,28 | -1.77 | 0.51 | 55.3 | -3.49 | 0.02 |
| 18,BPA,18 - 28,control,28 | -1.06 | 0.47 | 55.3 | -2.27 | 0.33 |
| 18,BPA,18 - 18,BPA,28 | -1.09 | 0.26 | 41 | -4.22 | 0.003 |
| 18,BPA,18 - 28,BPA,28 | -1.91 | 0.47 | 55.3 | -4.07 | 0.004 |
| 28,BPA,18 - 18,control,28 | -1.47 | 0.51 | 55.3 | -2.90 | 0.09 |
| 28,BPA,18 - 28,control,28 | -0.76 | 0.47 | 55.3 | -1.62 | 0.74 |
| 28,BPA,18 - 18,BPA,28 | -0.79 | 0.47 | 55.3 | -1.68 | 0.70 |
| 28,BPA,18 - 28,BPA,28 | -1.61 | 0.26 | 41 | -6.20 | <.0001 |
| 18,control,28 - 28,control,28 | 0.71 | 0.51 | 55.3 | 1.40 | 0.86 |
| 18,control,28 - 18,BPA,28 | 0.68 | 0.51 | 55.3 | 1.34 | 0.88 |
| 18,control,28 - 28,BPA,28 | -0.14 | 0.51 | 55.3 | -0.27 | 1.00 |
| 28,control,28 - 18,BPA,28 | -0.03 | 0.47 | 55.3 | -0.06 | 1.00 |
| 28,control,28 - 28,BPA,28 | -0.85 | 0.47 | 55.3 | -1.80 | 0.62 |
| 18,BPA,28 - 28,BPA,28 | -0.82 | 0.47 | 55.3 | -1.74 | 0.66 |
| **BPF** | | | | | |
| **contrast** | **estimate** | **SE** | **df** | **t.ratio** | **p.value** |
| 18,control,18 - 28,control,18 - | 0.25 | 0.34 | 68.2 | -0.73 | 1.00 |
| 18,control,18 - 18,BPF,18 | -0.34 | 0.34 | 68.2 | -1.00 | 0.97 |
| 18,control,18 - 28,BPF,18 | 0.00 | 0.34 | 68.2 | -0.01 | 1.00 |
| 18,control,18 - 18,control,28 | -1.84 | 0.23 | 44 | -7.88 | <.0001 |
| 18,control,18 - 28,control,28 | -2.18 | 0.34 | 68.2 | -6.36 | <.0001 |
| 18,control,18 - 18,BPF,28 | -2.19 | 0.34 | 68.2 | -6.38 | <.0001 |
| 18,control,18 - 28,BPF,28 | -1.60 | 0.34 | 68.2 | -4.67 | 0.0004 |
| 28,control,18 - 18,BPF,18 | -0.09 | 0.34 | 68.2 | -0.27 | 1.00 |
| 28,control,18 - 28,BPF,18 | 0.25 | 0.34 | 68.2 | 0.72 | 1.00 |
| 28,control,18 - 18,control,28 | -1.58 | 0.34 | 68.2 | -4.62 | 0.00 |
| 28,control,18 - 28,control,28 | -1.93 | 0.23 | 44 | -8.29 | <.0001 |
| 28,control,18 - 18,BPF,28 | -1.93 | 0.34 | 68.2 | -5.64 | <.0001 |
| 28,control,18 - 28,BPF,28 | -1.35 | 0.34 | 68.2 | -3.94 | 0.005 |
| 18,BPF,18 - 28,BPF,18 | 0.34 | 0.34 | 68.2 | 0.99 | 0.97 |
| 18,BPF,18 - 18,control,28 | -1.49 | 0.34 | 68.2 | -4.35 | 0.001 |
| 18,BPF,18 - 28,control,28 | -1.84 | 0.34 | 68.2 | -5.36 | <.0001 |
| 18,BPF,18 - 18,BPF,28 | -1.84 | 0.23 | 44 | -7.91 | <.0001 |
| 18,BPF,18 - 28,BPF,28 | -1.26 | 0.34 | 68.2 | -3.67 | 0.01 |
| 28,BPF,18 - 18,control,28 | -1.83 | 0.34 | 68.2 | -5.35 | <.0001 |
| 28,BPF,18 - 28,control,28 | -2.18 | 0.34 | 68.2 | -6.35 | <.0001 |
| 28,BPF,18 - 18,BPF,28 | -2.18 | 0.34 | 68.2 | -6.37 | <.0001 |
| 28,BPF,18 - 28,BPF,28 | -1.60 | 0.23 | 44 | -6.87 | <.0001 |
| 18,control,28 - 28,control,28 | -0.34 | 0.34 | 68.2 | -1.01 | 0.97 |
| 18,control,28 - 18,BPF,28 | -0.35 | 0.34 | 68.2 | -1.02 | 0.97 |
| 18,control,28 - 28,BPF,28 | 0.23 | 0.34 | 68.2 | 0.68 | 1.00 |
| 28,control,28 - 18,BPF,28 | -0.01 | 0.34 | 68.2 | -0.02 | 1.00 |
| 28,control,28 - 28,BPF,28 | 0.58 | 0.34 | 68.2 | 1.69 | 0.70 |
| 18,BPF,28 - 28,BPF,28 | 0.58 | 0.34 | 68.2 | 1.70 | 0.69 |
| **BPS** | | | | | |
| **contrast** | **estimate** | **SE** | **df** | **t.ratio** | **p.value** |
| 18,control,18 - 28,control,18 | 0.33 | 0.37 | 70.2 | 0.92 | 0.98 |
| 18,control,18 - 18,BPS,18 | 0.91 | 0.37 | 70.2 | 2.49 | 0.22 |
| 18,control,18 - 28,BPS,18 | 0.73 | 0.37 | 70.2 | 2.00 | 0.49 |
| 18,control,18 - 18,control,28 | -0.92 | 0.26 | 44 | -3.56 | 0.02 |
| 18,control,18 - 28,control,28 | -0.96 | 0.37 | 70.2 | -2.62 | 0.17 |
| 18,control,18 - 18,BPS,28 | 0.20 | 0.37 | 70.2 | 0.54 | 1.00 |
| 18,control,18 - 28,BPS,28 | -0.21 | 0.37 | 70.2 | -0.59 | 1.00 |
| 28,control,18 - 18,BPS,18 | 0.58 | 0.37 | 70.2 | 1.58 | 0.76 |
| 28,control,18 - 28,BPS,18 | 0.40 | 0.37 | 70.2 | 1.08 | 0.96 |
| 28,control,18 - 18,control,28 | -1.25 | 0.37 | 70.2 | -3.43 | 0.02 |
| 28,control,18 - 28,control,28 | -1.29 | 0.26 | 44 | -5.02 | 0.0002 |
| 28,control,18 - 18,BPS,28 | -0.14 | 0.37 | 70.2 | -0.38 | 1.00 |
| 28,control,18 - 28,BPS,28 | -0.55 | 0.37 | 70.2 | -1.50 | 0.80 |
| 18,BPS,18 - 28,BPS,18 | -0.18 | 0.37 | 70.2 | -0.49 | 1.00 |
| 18,BPS,18 - 18,control,28 | -1.83 | 0.37 | 70.2 | -5.00 | 0.0001 |
| 18,BPS,18 - 28,control,28 | -1.87 | 0.37 | 70.2 | -5.12 | 0.0001 |
| 18,BPS,18 - 18,BPS,28 | -0.71 | 0.26 | 44 | -2.77 | 0.13 |
| 18,BPS,18 - 28,BPS,28 | -1.13 | 0.37 | 70.2 | -3.08 | 0.06 |
| 28,BPS,18 - 18,control,28 | -1.65 | 0.37 | 70.2 | -4.51 | 0.0006 |
| 28,BPS,18 - 28,control,28 | -1.69 | 0.37 | 70.2 | -4.62 | 0.0004 |
| 28,BPS,18 - 18,BPS,28 | -0.53 | 0.37 | 70.2 | -1.46 | 0.83 |
| 28,BPS,18 - 28,BPS,28 | -0.94 | 0.26 | 44 | -3.67 | 0.01 |
| 18,control,28 - 28,control,28 | -0.04 | 0.37 | 70.2 | -0.11 | 1.00 |
| 18,control,28 - 18,BPS,28 | 1.11 | 0.37 | 70.2 | 3.05 | 0.06 |
| 18,control,28 - 28,BPS,28 | 0.70 | 0.37 | 70.2 | 1.92 | 0.54 |
| 28,control,28 - 18,BPS,28 | 1.15 | 0.37 | 70.2 | 3.16 | 0.05 |
| 28,control,28 - 28,BPS,28 | 0.74 | 0.37 | 70.2 | 2.03 | 0.47 |
| 18,BPS,28 - 28,BPS,28 | -0.41 | 0.37 | 70.2 | -1.13 | 0.95 |

Table S3. Summary statistics from the LDH activity estimated marginal means pairwise comparison for BPA, BPF, BPS. Names were ordered as acclimation temperature (18/28 °C), exposure (control/exposed), and acute temperature (18/28 °C).

| **BPA** | | | | | |
| --- | --- | --- | --- | --- | --- |
| **contrast** | **estimate** | **SE** | **df** | **t.ratio** | **p.value** |
| 18,control,18 - 28,control,18 | 7.29 | 10.73 | 64 | 0.68 | 1.00 |
| 18,control,18 - 18,BPA,18 | 3.05 | 10.73 | 64 | 0.28 | 1.00 |
| 18,control,18 - 28,BPA,18 | -10.57 | 10.73 | 64 | -0.99 | 0.98 |
| 18,control,18 - 18,control,28 | -29.19 | 7.87 | 41 | -3.71 | 0.01 |
| 18,control,18 - 28,control,28 | -24.02 | 10.73 | 64 | -2.24 | 0.34 |
| 18,control,18 - 18,BPA,28 | -13.20 | 10.73 | 64 | -1.23 | 0.92 |
| 18,control,18 - 28,BPA,28 | -45.38 | 10.73 | 64 | -4.23 | 0.002 |
| 28,control,18 - 18,BPA,18 | -4.24 | 9.93 | 64 | -0.43 | 1.00 |
| 28,control,18 - 28,BPA,18 | -17.86 | 9.93 | 64 | -1.80 | 0.62 |
| 28,control,18 - 18,control,28 | -36.48 | 10.73 | 64 | -3.40 | 0.02 |
| 28,control,18 - 28,control,28 | -31.31 | 6.81 | 41 | -4.60 | 0.001 |
| 28,control,18 - 18,BPA,28 | -20.50 | 9.93 | 64 | -2.06 | 0.45 |
| 28,control,18 - 28,BPA,28 | -52.67 | 9.93 | 64 | -5.30 | <.0001 |
| 18,BPA,18 - 28,BPA,18 | -13.62 | 9.93 | 64 | -1.37 | 0.87 |
| 18,BPA,18 - 18,control,28 | -32.24 | 10.73 | 64 | -3.01 | 0.07 |
| 18,BPA,18 - 28,control,28 | -27.07 | 9.93 | 64 | -2.73 | 0.13 |
| 18,BPA,18 - 18,BPA,28 | -16.25 | 6.81 | 41 | -2.39 | 0.28 |
| 18,BPA,18 - 28,BPA,28 | -48.43 | 9.93 | 64 | -4.88 | 0.0002 |
| 28,BPA,18 - 18,control,28 | -18.62 | 10.73 | 64 | -1.74 | 0.66 |
| 28,BPA,18 - 28,control,28 | -13.45 | 9.93 | 64 | -1.36 | 0.87 |
| 28,BPA,18 - 18,BPA,28 | -2.64 | 9.93 | 64 | -0.27 | 1.00 |
| 28,BPA,18 - 28,BPA,28 | -34.81 | 6.81 | 41 | -5.11 | 0.0002 |
| 18,control,28 - 28,control,28 | 5.17 | 10.73 | 64 | 0.48 | 1.00 |
| 18,control,28 - 18,BPA,28 | 15.98 | 10.73 | 64 | 1.49 | 0.81 |
| 18,control,28 - 28,BPA,28 | -16.19 | 10.73 | 64 | -1.51 | 0.80 |
| 28,control,28 - 18,BPA,28 | 10.81 | 9.93 | 64 | 1.09 | 0.96 |
| 28,control,28 - 28,BPA,28 | -21.36 | 9.93 | 64 | -2.15 | 0.39 |
| 18,BPA,28 - 28,BPA,28 | -32.18 | 9.93 | 64 | -3.24 | 0.04 |
| **BPF** | | | | | |
| **contrast** | **estimate** | **SE** | **df** | **t.ratio** | **p.value** |
| 18,control,18 - 28,control,18 | -0.54 | 7.01 | 77.5 | -0.08 | 1.00 |
| 18,control,18 - 18,BPF,18 | 11.17 | 7.01 | 77.5 | 1.59 | 0.75 |
| 18,control,18 - 28,BPF,18 | 4.34 | 7.01 | 77.5 | 0.62 | 1.00 |
| 18,control,18 - 18,control,28 | -34.97 | 5.58 | 44 | -6.27 | <.0001 |
| 18,control,18 - 28,control,28 | -27.02 | 7.01 | 77.5 | -3.85 | 0.01 |
| 18,control,18 - 18,BPF,28 | -19.81 | 7.01 | 77.5 | -2.83 | 0.10 |
| 18,control,18 - 28,BPF,28 | -41.18 | 7.01 | 77.5 | -5.88 | <.0001 |
| 28,control,18 - 18,BPF,18 | 11.71 | 7.01 | 77.5 | 1.67 | 0.71 |
| 28,control,18 - 28,BPF,18 | 4.88 | 7.01 | 77.5 | 0.70 | 1.00 |
| 28,control,18 - 18,control,28 | -34.43 | 7.01 | 77.5 | -4.91 | 0.0001 |
| 28,control,18 - 28,control,28 | -26.47 | 5.58 | 44 | -4.75 | 0.0005 |
| 28,control,18 - 18,BPF,28 | -19.27 | 7.01 | 77.5 | -2.75 | 0.12 |
| 28,control,18 - 28,BPF,28 | -40.63 | 7.01 | 77.5 | -5.80 | <.0001 |
| 18,BPF,18 - 28,BPF,18 | -6.83 | 7.01 | 77.5 | -0.97 | 0.98 |
| 18,BPF,18 - 18,control,28 | -46.14 | 7.01 | 77.5 | -6.58 | <.0001 |
| 18,BPF,18 - 28,control,28 | -38.18 | 7.01 | 77.5 | -5.45 | <.0001 |
| 18,BPF,18 - 18,BPF,28 | -30.98 | 5.58 | 44 | -5.56 | <.0001 |
| 18,BPF,18 - 28,BPF,28 | -52.34 | 7.01 | 77.5 | -7.47 | <.0001 |
| 28,BPF,18 - 18,control,28 | -39.31 | 7.01 | 77.5 | -5.61 | <.0001 |
| 28,BPF,18 - 28,control,28 | -31.35 | 7.01 | 77.5 | -4.47 | 0.0007 |
| 28,BPF,18 - 18,BPF,28 | -24.15 | 7.01 | 77.5 | -3.45 | 0.02 |
| 28,BPF,18 - 28,BPF,28 | -45.52 | 5.58 | 44 | -8.16 | <.0001 |
| 18,control,28 - 28,control,28 | 7.95 | 7.01 | 77.5 | 1.14 | 0.95 |
| 18,control,28 - 18,BPF,28 | 15.16 | 7.01 | 77.5 | 2.16 | 0.39 |
| 18,control,28 - 28,BPF,28 | -6.21 | 7.01 | 77.5 | -0.89 | 0.99 |
| 28,control,28 - 18,BPF,28 | 7.21 | 7.01 | 77.5 | 1.03 | 0.97 |
| 28,control,28 - 28,BPF,28 | -14.16 | 7.01 | 77.5 | -2.02 | 0.48 |
| 18,BPF,28 - 28,BPF,28 | -21.37 | 7.01 | 77.5 | -3.05 | 0.06 |
| **BPS** | | | | | |
| **contrast** | **estimate** | **SE** | **df** | **t.ratio** | **p.value** |
| 18,control,18 - 28,control,18 | -5.06 | 7.51 | 64.9 | -0.67 | 1.00 |
| 18,control,18 - 18,BPS,18 | 9.34 | 7.51 | 64.9 | 1.24 | 0.92 |
| 18,control,18 - 28,BPS,18 | 2.33 | 7.51 | 64.9 | 0.31 | 1.00 |
| 18,control,18 - 18,control,28 | -31.10 | 4.76 | 44 | -6.53 | <.0001 |
| 18,control,18 - 28,control,28 | -42.47 | 7.51 | 64.9 | -5.66 | <.0001 |
| 18,control,18 - 18,BPS,28 | -20.42 | 7.51 | 64.9 | -2.72 | 0.14 |
| 18,control,18 - 28,BPS,28 | -32.77 | 7.51 | 64.9 | -4.36 | 0.001 |
| 28,control,18 - 18,BPS,18 | 14.40 | 7.51 | 64.9 | 1.92 | 0.54 |
| 28,control,18 - 28,BPS,18 | 7.39 | 7.51 | 64.9 | 0.98 | 0.98 |
| 28,control,18 - 18,control,28 | -26.04 | 7.51 | 64.9 | -3.47 | 0.02 |
| 28,control,18 - 28,control,28 | -37.41 | 4.76 | 44 | -7.85 | <.0001 |
| 28,control,18 - 18,BPS,28 | -15.36 | 7.51 | 64.9 | -2.05 | 0.46 |
| 28,control,18 - 28,BPS,28 | -27.71 | 7.51 | 64.9 | -3.69 | 0.01 |
| 18,BPS,18 - 28,BPS,18 | -7.01 | 7.51 | 64.9 | -0.93 | 0.98 |
| 18,BPS,18 - 18,control,28 | -40.44 | 7.51 | 64.9 | -5.39 | <.0001 |
| 18,BPS,18 - 28,control,28 | -51.81 | 7.51 | 64.9 | -6.90 | <.0001 |
| 18,BPS,18 - 18,BPS,28 | -29.76 | 4.76 | 44 | -6.25 | <.0001 |
| 18,BPS,18 - 28,BPS,28 | -42.11 | 7.51 | 64.9 | -5.61 | <.0001 |
| 28,BPS,18 - 18,control,28 | -33.43 | 7.51 | 64.9 | -4.45 | 0.001 |
| 28,BPS,18 - 28,control,28 | -44.80 | 7.51 | 64.9 | -5.97 | <.0001 |
| 28,BPS,18 - 18,BPS,28 | -22.75 | 7.51 | 64.9 | -3.03 | 0.07 |
| 28,BPS,18 - 28,BPS,28 | -35.10 | 4.76 | 44 | -7.37 | <.0001 |
| 18,control,28 - 28,control,28 | -11.37 | 7.51 | 64.9 | -1.51 | 0.80 |
| 18,control,28 - 18,BPS,28 | 10.68 | 7.51 | 64.9 | 1.42 | 0.84 |
| 18,control,28 - 28,BPS,28 | -1.67 | 7.51 | 64.9 | -0.22 | 1.00 |
| 28,control,28 - 18,BPS,28 | 22.05 | 7.51 | 64.9 | 2.94 | 0.08 |
| 28,control,28 - 28,BPS,28 | 9.70 | 7.51 | 64.9 | 1.29 | 0.90 |
| 18,BPS,28 - 28,BPS,28 | -12.35 | 7.51 | 64.9 | -1.65 | 0.72 |

## Supplementary figures


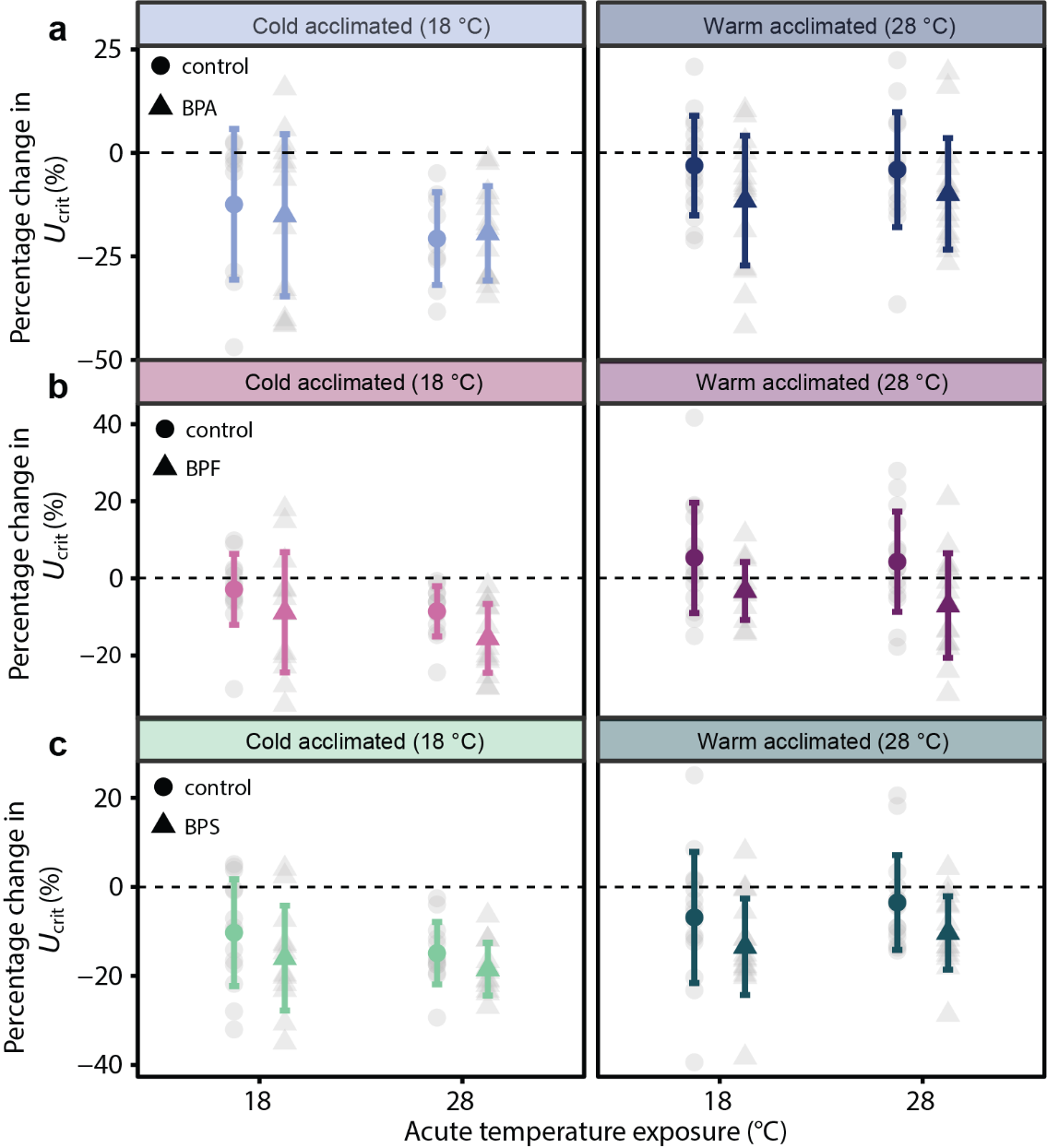


Fig. S1. Percentage change in post-exposure *U*_crit_ (3 weeks cold/warm with bisphenol exposure) from pre-exposure (prior to exposure). Change in *U*_crit_ in the bisphenol A (a), bisphenol F (b), and bisphenol S (c) treatment exposed at 18 and 28 °C acute temperature between the cold- (left plot) and warm-acclimated (right plot) treatments. Positive values indicate an increase in *U*_crit_ or *Q*_10_, and negative values indicate a decrease in in *U*_crit_. Data was presented as mean ± s.d. (*n* = 9–15) with individual data as grey points.
